# HCC-derived CX3CL1 affects hepatocellular carcinoma prognosis and CX3CR1 + MDSC infiltration

**DOI:** 10.1186/s40001-025-02410-z

**Published:** 2025-03-06

**Authors:** Xiaoling Zhang, Yidan Lou, Song Zheng, Xu Chang

**Affiliations:** 1 Department of Medical Oncology, Hangzhou First People’s Hospital, Hangzhou, 310006 China; 2 Zhejiang University School of Medicine, Hangzhou, 310006 China; 3 Key Laboratory of Clinical Cancer Pharmacology and Toxicology Research of Zhejiang Province, Hangzhou First People’s Hospital, Hangzhou, 310006 China; 4 Department of Interventional Therapy II, Shandong Cancer Hospital and Institute, Shandong First Medical University and Shandong Academy of Medical Sciences, Jinan, Shandong China

**Keywords:** CX3CL1, HCC, MDSC, Hepatocellular carcinoma, CX3CR1

## Abstract

**Background:**

Hepatocellular carcinoma (HCC) remains a leading cause of cancer-related mortality worldwide, largely because of its ability to reshape the tumor microenvironment and evade immune surveillance.

**Methods:**

CX3CL1 expression in HCC tumor tissues was assessed via immunohistochemistry, while plasma levels were quantified using enzyme-linked immunosorbent assays (ELISAs). CX3CR1-positive immune cell infiltration was analyzed by immunofluorescence. The associations among CX3CL1 expression, CX3CR1-positive cell infiltration, and patient prognosis were examined. Additionally, cell-based assays were conducted to evaluate the impact of CX3CL1 amplification on the expression of myeloid-derived suppressor cell (MDSC)-recruiting factors.

**Results:**

Elevated CX3CL1 levels were significantly correlated with increased MDSC infiltration in the tumor microenvironment and poorer patient prognosis. CX3CL1 amplification led to the upregulation of MDSC-recruiting factors, suggesting a potential mechanism for immune evasion.

**Conclusions:**

These findings highlight the possible involvement of CX3CL1 in HCC progression via MDSC recruitment, suggesting that it is a promising therapeutic target for promoting antitumor immunity. Further studies are needed to confirm these findings and explore potential therapeutic strategies.

**Supplementary Information:**

The online version contains supplementary material available at 10.1186/s40001-025-02410-z.

## Introduction

Hepatocellular carcinoma (HCC) is the sixth most common malignant tumor globally and a major cause of cancer-related mortality [1]. Surgical resection, liver transplantation, chemotherapy, and tyrosine kinase inhibitors (TKIs) offer limited effectiveness in treating HCC, with radical resection rates below 30% and recurrence rates exceeding 50% [2, 3]. The advent of immune checkpoint inhibitors has substantially advanced HCC management [4]. Local regional therapies, such as radiofrequency ablation (RFA) and transarterial chemoembolization (TACE), also play crucial roles in managing HCC during the early to intermediate stages, accounting for 50%−60% of treatment approaches [5].

Chemokines, categorized into CXC, CC, C, and CX3C groups, are integral to tumor-associated inflammation and progression and influence leukocyte recruitment and tumor cell metastasis [6]. Notably, CX3C chemokine ligand 1 (CX3CL1), or fractalkine produced by endothelial cells, binds exclusively to CX3C chemokine receptor 1 (CX3CR1), a G protein-coupled receptor on CD8 + T lymphocytes, NK cells, and monocytes. Its expression strongly impacts the prognosis of various cancers, including HCC. For example, HCC-derived CX3CL1 facilitates tumor infiltration and induces apoptosis [7]. Additionally, the microRNA miR-561-5p negatively regulates CX3CL1 and CX3CR1 levels in NK cells, thereby influencing antitumor activities [8]. However, the complete role of CX3CL1 in HCC and its interactions with myeloid-derived suppressor cells (MDSCs) are not fully understood. CX3CL1 was shown to mediate the migration of MDSCs, especially monocytic MDSCs (M-MDSCs) [9].

Recent advances have shed light on the role of the immune system in the pathophysiology of tumors and inflammation, especially through the study of CX3CR1-positive immune cells [10, 11]. These cells, including macrophages, dendritic cells, and certain T cells, are crucial in conditions such as tumors and inflammatory diseases [12–14] because they modulate immune responses, either by promoting tumor growth through immune suppression or contributing to inflammation through cytokine production and antigen presentation. MDSCs play a pivotal role in the tumor-associated immunosuppressive environment, with subsets including polymorphonuclear (PMN-MDSCs) and monocytic (M-MDSCs) MDSCs [15, 16]. Cluster analysis revealed distinct chemokine gene expression between these subsets, with M-MDSCs exhibiting a broader profile, including elevated levels of genes such as Cx3cr1 [17]. The accumulation of MDSCs in HCC is associated with tumor progression, immune suppression, and poor patient prognosis, including larger tumors, lymph node metastasis, and early recurrence [18, 19]. Moreover, factors such as interferon-alpha (IFN-α) and hypoxia-inducible factor 1-alpha (HIF-1α) are known to regulate MDSC recruitment through the CX3CL1-CX3CR1 axis, further influencing antitumor responses [9]. Several chemokines and signaling molecules, such as CXCL10, CXCL13, CXCL5, CXCL2, DDR2, and STAT3, have been shown to modulate the tumor microenvironment by facilitating MDSC recruitment and accumulation [20–22]. Understanding these mechanisms is critical for the development of new therapeutic strategies that target MDSC-mediated immune evasion.

This study aimed to investigate the correlation between CX3CL1 expression and CX3CR1-positive MDSC infiltration, as well as its impact on the prognosis of HCC patients. By analyzing CX3CL1 expression in HCC tissues using immunohistochemistry, assessing CX3CR1-positive immune cells through immunofluorescence, and measuring preoperative plasma CX3CL1 levels using enzyme-linked immunosorbent assays (ELISAs) in patients undergoing TACE, this research provides insights into the role of CX3CL1 in HCC progression and offers a foundation for potential therapeutic interventions targeting the CX3CL1‒CX3CR1 axis.

## Methods

### Sample collection and selection

A total of 59 hepatocellular cancer biopsy samples were collected from two centers: the Department of Interventional Therapy at Shandong Cancer Hospital and the Department of Oncology at Hangzhou First People's Hospital. These samples were obtained through percutaneous biopsy procedures performed on patients diagnosed with HCC. All samples were anonymized to ensure patient privacy. This study was conducted according to the principles of the Declaration of Helsinki and was approved by the Institutional Review Boards of both Shandong Cancer Hospital and Hangzhou First People's Hospital. Written consent was obtained from each patient.

### Cell culture

The PLC/PRF/5, HepG2, and Lo2 cell lines were cultured in Dulbecco's modified Eagle’s medium (DMEM) supplemented with 10% fetal bovine serum (FBS) and 1% penicillin‒streptomycin. The cells were maintained at 37 °C in a humidified atmosphere containing 5% CO_2_. The culture medium was changed every 2‒3 days, and the cells were passaged at 80‒90% confluence via 0.25% trypsin–EDTA.

### Immunohistochemistry for CX3CL1 expression

Immunohistochemistry (IHC) was used to assess CX3CL1 expression in HCC samples. Immunoreactivity was primarily observed in tumor tissues and adjacent liver tissues from biopsy samples. Fifty-nine patients were included in the immunohistochemical study. The primary antibody used for CX3CL1 detection was purchased from Proteintech (Cat No. 10108–2-AP; Wuhan, China). The samples were fixed, embedded in paraffin, and sectioned. Following deparaffinization and rehydration, antigen retrieval was conducted using a citrate buffer (pH 6.0). After nonspecific binding sites were blocked, the slides were incubated with the primary antibody, followed by incubation with an HRP-conjugated secondary antibody. Diaminobenzidine (DAB) was used as the chromogen, and the sections were counterstained with hematoxylin. The evaluation of CX3CL1 expression was standardized using an immunoreactive score (SI), which was determined by the staining index (SI). Two independent pathologists, blinded to the histopathological and clinical data, evaluated CX3CL1 expression using the staining index (SI) method. The SI for each sample was calculated by multiplying the stain intensity (0 = negative, 1 = canary yellow, 2 = claybank, and 3 = brown) by the percentage of positive cells (1 = less than 25%, 2 = 25–50%, 3 = 51–75%, and 4 = more than 75%) [23]. SI scores ranged from 1 to 6 for low expression and 9 to 16 for high expression.

### Immunofluorescence analysis

Immunofluorescence was performed to assess the prevalence of CX3CR1-positive immune cells in samples exhibiting low and high CX3CL1 expression. A subset of 23 samples, 11 with low and 12 with high CX3CL1 expression, was analyzed on the basis of the IHC results. The preparatory procedures mirrored those used for IHC. After antigen retrieval and blocking, the sections were incubated with fluorescently labeled antibodies. Visualization and quantification of CX3CR1^+^ immune cells, including CX3CR1^+^CD8^+^, CX3CR1^+^F4/80^+^, CX3CR1^+^Ly6G/Ly6C^+^, and CX3CR1^+^NK1.1^+^ subtypes, were performed using fluorescence microscopy. Total cell counts were determined for each field, followed by identification and quantification of CX3CR1^+^Ly6G/Ly6C + cells, expressed as a percentage of total cells. Antibodies specific for CX3CR1 and other markers were obtained from Novus Biologicals (USA) and Proteintech. The antibodies used included CX3CR1 (#NBP1-76,949, Novus Biologicals, USA), F4/80 (#NB600-404SS, Novus Biologicals, USA), CD8 (#NBP1-49045SS, Novus Biologicals, USA), CD161/NK1.1 (#NB100-77528SS, Novus Biologicals, USA), and Ly-6G/Ly-6C (#NBP2-00441SS, Novus Biologicals, USA).

### Blood ELISAs

Serum samples from 110 HCC patients collected before TACE were analyzed using ELISAs to determine the baseline CX3CL1 concentration. After collection, the serum CX3CL1 concentrations were quantitatively analyzed using a specific enzyme-linked immunosorbent assay (ELISA) kit (#E-EL-H0044c, Elabscience, Wuhan, China) according to the manufacturer's instructions. This method involves the preparation of standards and patient serum samples, their application to ELISA plates precoated with an anti-CX3CL1 antibody, and subsequent detection with a horseradish peroxidase (HRP)-conjugated secondary antibody. The reaction was visualized with a substrate solution, and the optical density was measured using a microplate reader. The concentration of CX3CL1 in the serum samples was then determined by comparing the absorbance of the samples to a standard curve.

### Plasmid extraction and transfection

The pCMV-CX3CL1 plasmid (#P41525, MiaoLing Biology, China) was amplified with the EndoFree Plasmid Midi Kit (#CW21055) following the manufacturer’s protocol. Transfection of target cells was performed using the JetPrime reagent (Polyplus-transfection) according to the manufacturer’s guidelines. Briefly, cells were seeded at 70–80% confluence in 6-well plates. The transfection mixture was prepared by combining plasmid DNA (2 µg) with JetPrime reagent (3 µL per µg of DNA) in JetPrime buffer. The mixture was then added dropwise to the cells and incubated for 24–72 h at 37 °C in a CO₂ incubator.

### Total RNA isolation and qRT‒PCR

Total RNA was isolated using the RNAprep Pure MicroKit (#RN07, Aidlab, Beijing, China). Genomic DNA contamination was eliminated using PC70-TRUEscript RT MasterMix. Quantitative real-time PCR (qRT‒PCR) was performed with Universal SYBR Green Fast qPCR Mix (#RK21203, China) on an ABI-7900HT Fast Real‒Time PCR System (Applied Biosystems). GAPDH was used as an internal control. Reactions were run in triplicate, and relative gene expression was calculated using the 2^‒ΔΔCt^ method. The primer sequences for CX3CL1, CXCL10, DDR2, STAT3, CXCL13, CXCL8, CXCL2, ARG-1, IFN-α, and GAPDH are provided in the Supplementary Table.

### ELISA for IFN-α and CXCL2 in cell supernatants

Supernatants from CX3CL1-amplified and untreated cells were collected. IFN-α and CXCL2 levels were measured via a Human IFNα ELISA Kit (#E-EL-H6123, Elabscience, China) and a Human GROβ/CXCL2 ELISA Kit (#E-EL-H1904, Elabscience, China), following the manufacturer’s instructions. The absorbance was read on a microplate reader, and the concentrations were determined from standard curves.

### Statistical analysis

SPSS software version 26.0 and R programming language were used to analyze the data, with a *p* value of < 0.05 considered statistically significant for comparing the differences in cell percentages between the two groups. Chi-square tests were used to examine the relationships between categorical variables. Survival curves were plotted using the Kaplan‒Meier method, and the statistical significance between groups was determined using the log-rank test.

## Results

### Distribution of CX3CR1^+^Ly6G/Ly6C^+^ cells in different expression groups

**Fig. 1:**
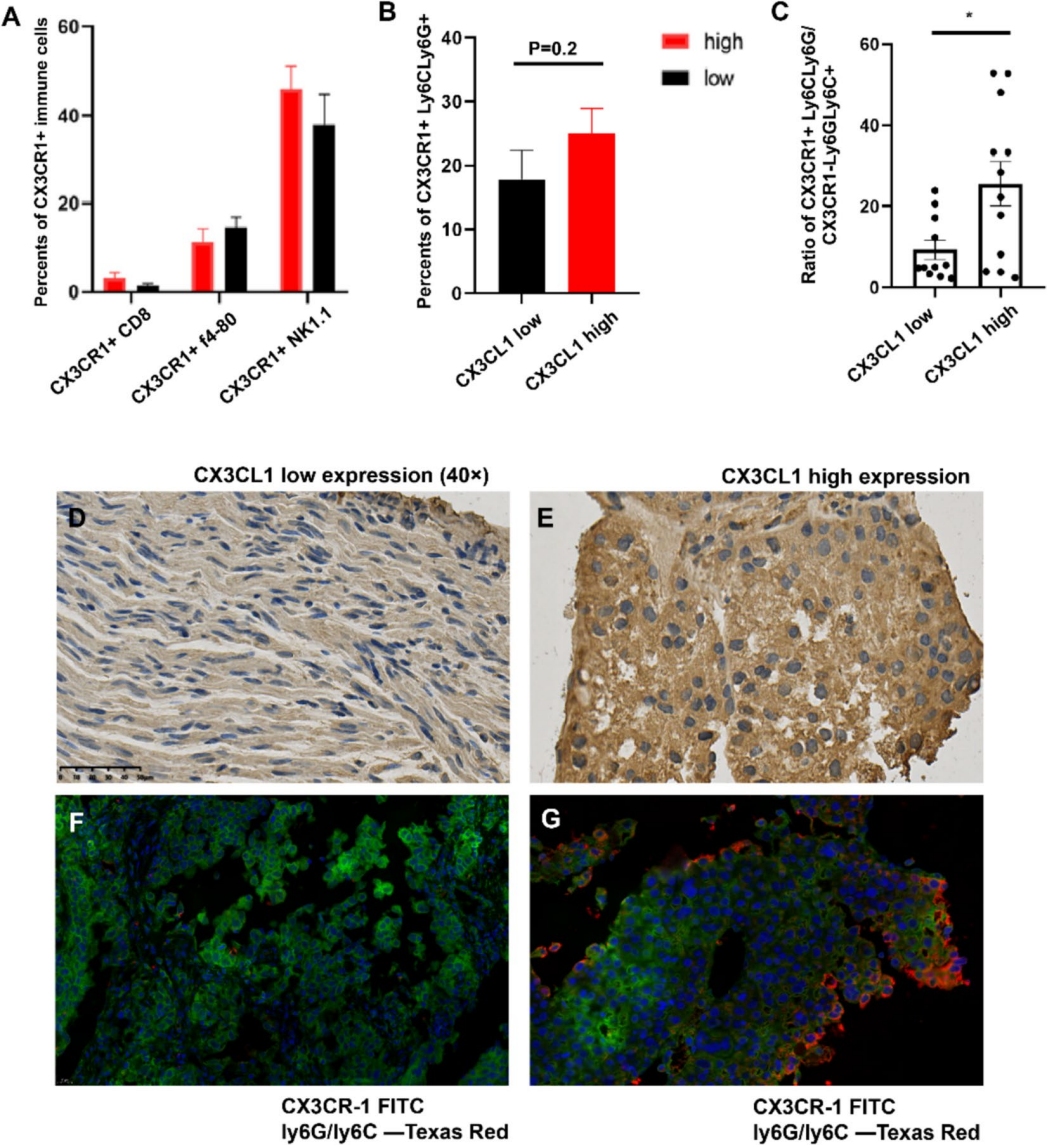
Immunofluorescence and immunohistochemical analyses of cellular markers in CX3CL1 expression groups. **A** Immunofluorescence assay comparing the counts of NK cells, macrophages, and CD8^+^ T cells in groups with differing levels of CX3CL1 expression. **B** Analysis of CX3CR^+^Ly6C/Ly6G^+^ MDSCs through immunofluorescence in groups with varying CX3CL1 expression levels. **C** Comparison of the ratio of CX3CR1^+^Ly6C/Ly6G^+^ to CX3CR^−^Ly6G/Ly6C^+^ cells in high versus low CX3CL1 expression groups. **D**, **E** High and low expressions of CX3CL1 and CXCR1 with Ly6G/Ly6C in HCC are represented, showing differences in staining intensity and distribution. **F**, **G** showed immunofluorescence analysis detecting CX3CR1^+^Ly6G/Ly6C^+^ cells in low and high expression samples

The expression of CX3CL1 was low in 26 patients (47.3%) and high in 33 patients (55.9%) according to the IHC scoring system. To assess the distribution of various immune cell types in the context of CX3CL1 expression, we first visualized the presence of MDSCs, NK cells, macrophages, and CD8^+^ T cells across the low- and high-expression groups using immunofluorescence. As shown in Fig. 1A, no significant differences were observed in the proportions of these immune cells between the two groups. The average number of CD8^+^ T cells was 3.29 in the high-expression group versus 1.53 in the low-expression group; the average number of macrophages was 11.49 versus 14.59; and the average number of NK cells was 46.02 versus 37.88.

Immunofluorescence analysis revealed a greater number of CX3CR^+^Ly6C/Ly6G^+^ MDSCs in the high CX3CL1 expression group than in the low-expression group, with mean counts of 25.05 versus 17.89 (*P* = 0.2, Fig. 1B). Additionally, we found a significant difference in the ratio of CX3CR1^+^Ly6C/Ly6G^+^ cells to CX3CR^−^Ly6G/Ly6C^+^ cells between the groups. The high-expression group (*N* = 12) presented a mean expression ratio of 25.58, whereas the mean expression ratio was 9.32 in the low-expression group (*N* = 11; Fig. 1C).

### CX3CL1-overexpressing HCC shows upregulation of MDSC-recruiting factors

**Fig. 2:**
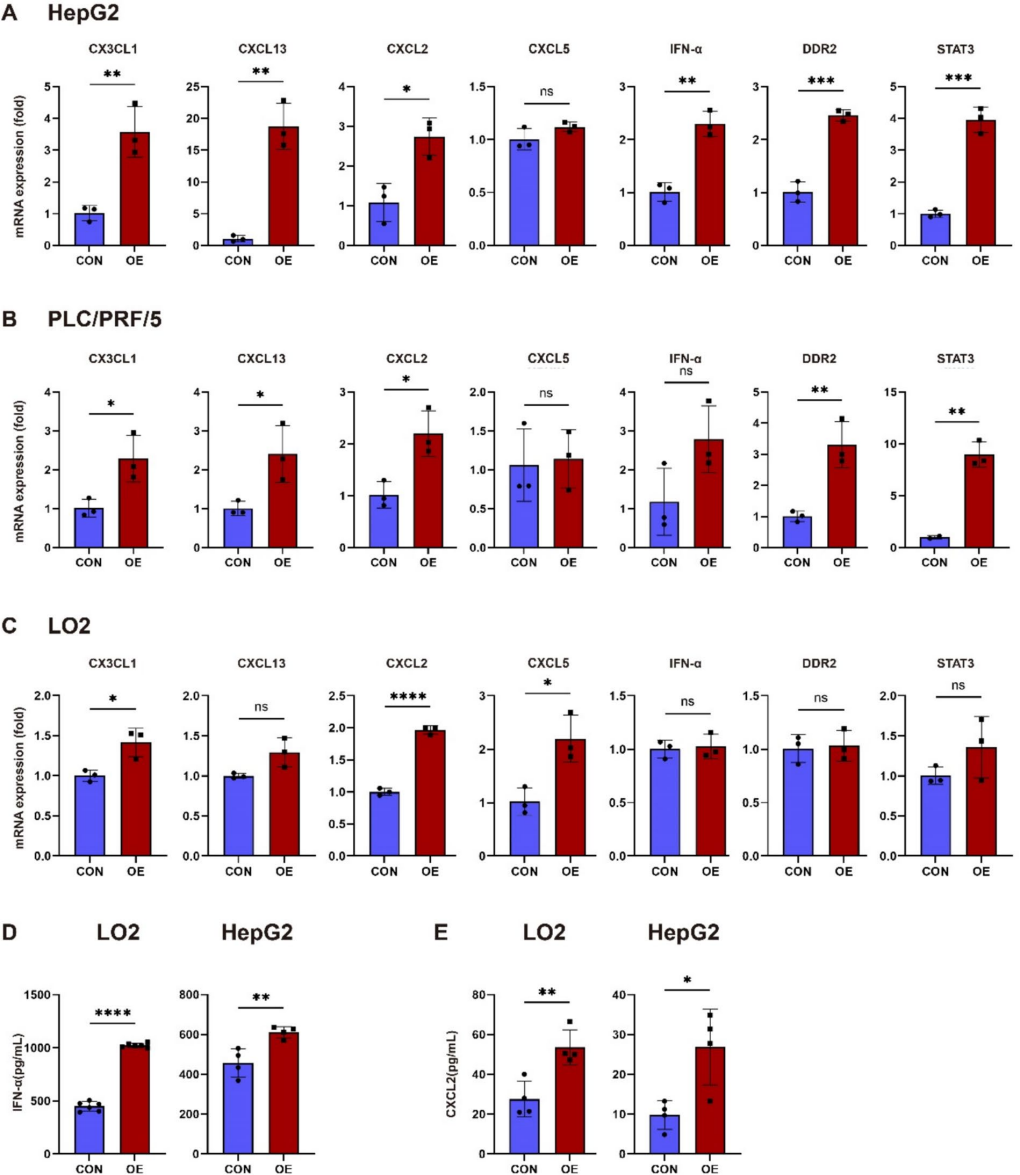
Effects of CX3CL1 overexpression on the expression levels of various genes in different cell lines. **A** HepG2 cells, **B** PLC/PRF/5 cells, **C** Lo2 cells. **A**–**C** present qPCR results for mRNA expression of CX3CL1, CXCL13, STAT3, DDR2, and other related genes, comparing the overexpression group (OE) with the control group (CON). Effects of CX3CL1 overexpression on IFN-α (**D**) and CXCL2 (**E**) secretion levels in cell supernatants, as measured by ELISA. The Y-axis shows the concentration (pg/ml). Data are presented as the mean ± SD from three independent experiments. *p < 0.05; **p < 0.01; ***p < 0.001; ****p < 0.0001

To further investigate the changes in MDSC-related recruiting factors following CX3CL1 overexpression, we overexpressed the target gene CX3CL1 in three HCC and normal hepatocyte cell lines—HepG2, PLC/PRF/5, and Lo2—via plasmid transfection. The cells were divided into an overexpression group (OE) and a control group (CON). qPCR analysis (Fig. 2A–C) revealed that CX3CL1 expression was significantly greater in the OE group than in the CON group across all three cell lines, confirming successful transfection. We subsequently assessed the expression levels of MDSC-recruiting chemokines. The results showed that CXCL2 was consistently upregulated in the OE group in all three cell lines. CXCL13, DDR2, and STAT3 were upregulated in the OE group in both the HepG2 and PLC/PRF/5 cell lines, CXCL5 was upregulated in the OE group in the Lo2 cell line, and IFN-α was upregulated in the OE group in the HepG2 cell line.

To quantify the levels of IFN-α and CXCL2 in the cell supernatants, we performed ELISAs. Supernatants from the OE and CON groups were collected post-transfection, and the ELISA results demonstrated that the IFN-α (Fig. 2D) and CXCL2 (Fig. 2E) levels were elevated in both the Lo2 and HepG2 cells in the OE group compared with those in the CON group.

### CX3CL1 expression detected by IHC predicts the survival of HCC patients

**Fig. 3:**
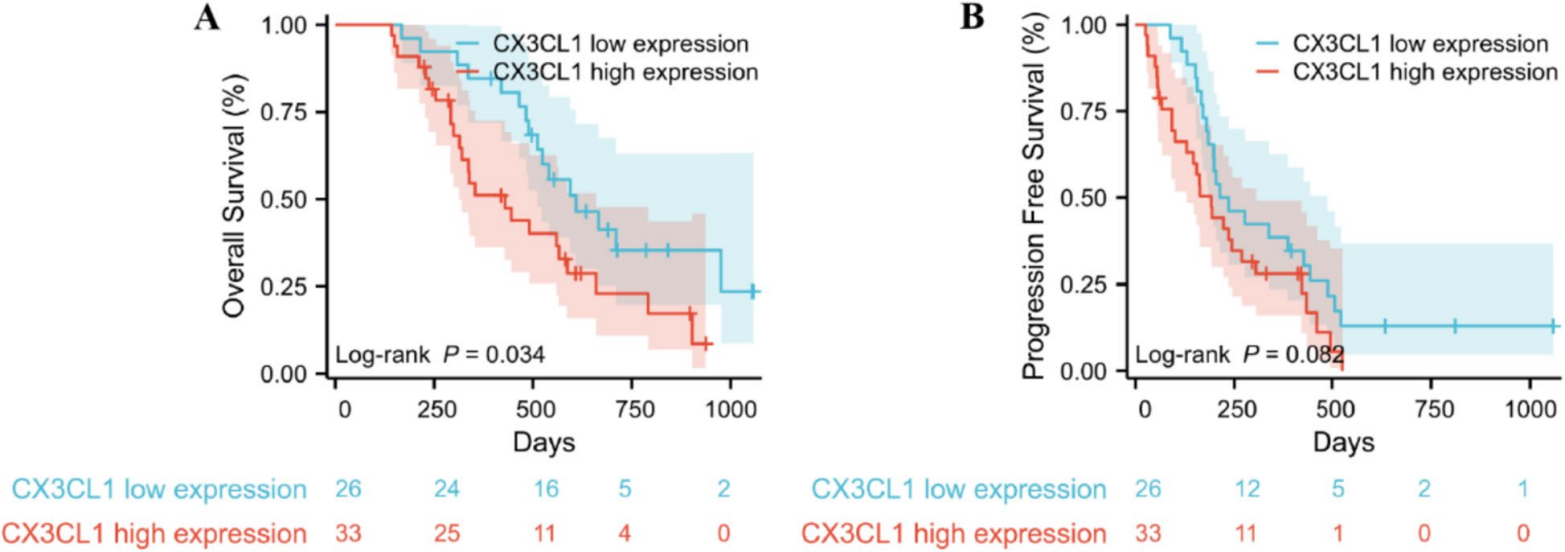
Survival analysis of HCC patients based on CX3CL1 expression. **A** Kaplan–Meier curves for OS comparing patients with low versus high CX3CL1 expression. **B** Kaplan–Meier curves for PFS in groups with differing levels of CX3CL1 expression

Progression-free survival (PFS) and overall survival (OS) were analyzed in the high- and low-expression cohorts in this study (Fig. 3A, B). Among them, CX3CL1 expression was significantly correlated with OS in hepatocellular carcinoma patients (low vs. high expression, 609 vs. 429 days, *P* = 0.0338), whereas there was no statistically significant difference in PFS (low vs. high expression, 225 vs. 192 days, *P* = 0.0825).

### Serum CX3CL1 concentration and its association with survival outcomes and clinicopathological findings in HCC patients receiving TACE

Given the observed correlation between CX3CL1 expression in tumor samples and patient survival, we expanded our sample size to examine this relationship in HCC patients receiving interventional therapy. The associations between clinicopathological factors and the concentration of serum CX3CL1 before TACE are detailed in Table 1. Receiver operating characteristic (ROC) curve analysis was employed to determine the cutoff value for the serum CX3CL1 concentration, which was established as 1209 pg/ml. In our serum analysis via ELISAs, 91 patients (82.7%) presented low CX3CL1 expression, and 19 (17.3%) presented high expression. No significant differences in Barcelona Clinic Liver Cancer (BCLC) stage, mean alpha-fetoprotein (AFP) level, vascular invasion, or distant metastasis were detected between the low- and high-expression groups, suggesting that CX3CL1 expression is not associated with these clinicopathological features. There was no correlation between AFP and CX3CL1 expression. With respect to treatment modalities, no significant differences were observed between the low-expression and high-expression groups. The majority of patients underwent combined treatment with TACE and targeted therapy, whereas a smaller proportion received TACE alone.

**Table 1:** Baseline characteristics of HCC patients undergoing TACE

Varies	CX3CL1 low expression (*n*, %)	CX3CL1 high expression (*n*, %)		*P*
BCLC				0.056
A	23 (25.3%)		0	
B	16 (17.6%)		6 (31.6%)	
C	49 (53.8%)		13 (68.4%)	
NED	3 (3.3%)		0	
AFP (mean, μg/L)	11622		115524	0.474
Vascular invasion				0.653
Yes	38 (41.8%)		9 (47.4%)	
No	53 (58.2%)		10 (52.6%)	
Distant metastasis				
Yes	26 (28.6%)		6 (31.6%)	0.793
No	65 (71.4%)		13 (68.4%)	
Treatment				0.338
TACE/TACE+targeted therapy	63 (69.2%)		11 (57.9%)	
TACE+targeted+immunotherapy	28 (30.8%)		9 (42.1%)	
Total	91		19	

**Fig. 4:**
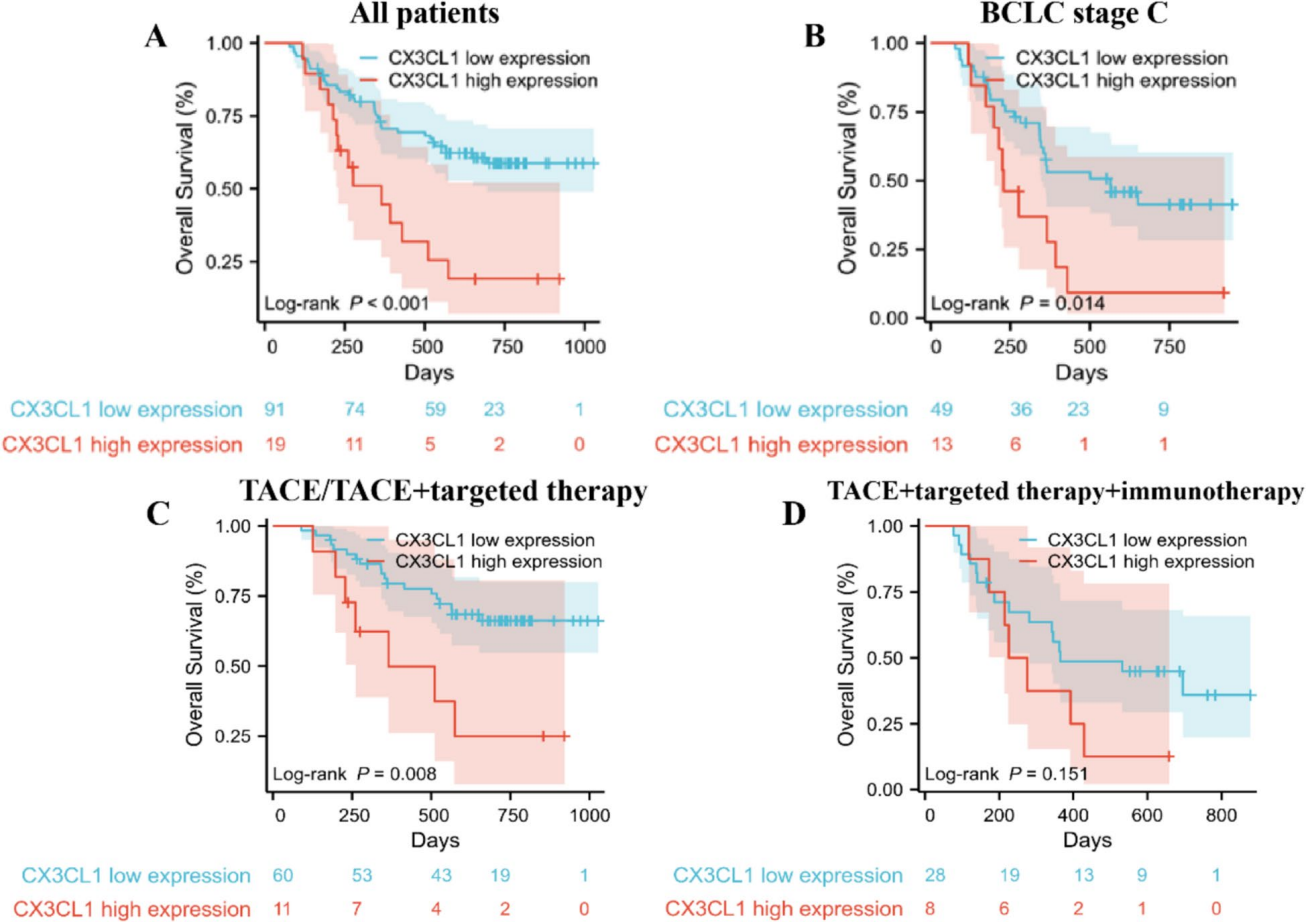
A comprehensive analysis of serum CX3CL1 expression in HCC patients and its association with prognosis. **A** Overall Survival for low vs. high CX3CL1 expression in 110 HCC patients. **B** Overall Survival for low vs. high CX3CL1 expression in patients at BCLC stage C subgroups. **C** Compared the OS curves in patients who received TACE or TACE and targeted therapy. **D** compared the OS curves in patients who received TACE plus both targeted therapy and immunotherapy

Our analysis revealed a distinct survival advantage associated with low CX3CL1 expression levels compared with high expression levels, indicating a significant improvement in OS (Fig. 4A, P < 0.001). Further delineation of the data based on the BCLC staging system, particularly for patients classified under stage C (Fig. 4B), demonstrated that individuals with low CX3CL1 expression exhibited a markedly prolonged median OS of 564 days, as opposed to the 290 days observed in their high expression counterparts (*P* = 0.014).

﻿Acknowledging the heterogeneity of treatment regimens and clinicopathological features, we performed a stratified survival analysis. Figure 4C and D show the OS curves for patients receiving TACE plus targeted therapy stratified by their receipt of immunotherapy. Notably, among patients who did not receive immunotherapy (*n* = 74), low CX3CL1 expression was associated with a significant survival benefit (*P* = 0.008). In contrast, among those who underwent immunotherapy (*N* = 36), the correlation between CX3CL1 expression and OS did not reach statistical significance (low vs. high expression, 365 vs*.* 225 days, *P* = 0.151).

## Discussion

Numerous studies have reported the upregulation of various chemokines and their receptors in HCC compared with the surrounding liver [24, 25]; however, the role of the chemokine system in cancer biology remains unclear [26]. Our research provides evidence of a correlation between the expression of CX3CL1 and the accumulation of CX3CR1 + MDSCs in HCC tissues. This relationship highlights the role of CX3CL1 as a key factor that recruits immunosuppressive cells to the tumor site through a CX3CR1-dependent mechanism, thus modulating the tumor microenvironment. Previous studies have shown elevated levels of MDSCs in the peripheral blood of HCC patients compared with healthy individuals or hepatitis patients [27], with a higher MDSC incidence linked to poor prognosis, early recurrence, and resistance to treatments such as radical resection and hepatic arterial infusion chemotherapy [27–30]. These findings suggest that MDSCs play a crucial role in HCC progression by promoting immune evasion. In our study, the presence of CX3CR1^+^ MDSCs in the tumor microenvironment, likely driven by CX3CL1, appeared to contribute to this immunosuppression, potentially facilitating tumor progression.

The finding that low levels of CX3CL1 expression in tumor and plasma samples are associated with improved overall survival rates in HCC patients is consistent with the hypothesis that reducing the recruitment of immunosuppressive MDSCs may alleviate immune evasion, thereby increasing the efficacy of antitumor responses. Furthermore, when the data were stratified on the basis of the BCLC staging system, particularly for patients classified under stage C, individuals with low CX3CL1 expression exhibited a markedly prolonged median OS. This trend was consistent even after considering the heterogeneity of treatment regimens and clinicopathological features.

Interestingly, the prognostic role of CX3CL1 varies across different cancers. In colon and gastric cancer, high CX3CL1 expression is associated with a favorable prognosis, primarily through its recruitment of CD8^+^ T cells and natural killer (NK) cells, which promote antitumor immunity [31–33]. However, in breast cancer and HCC, increased CX3CL1 expression has been linked to poor outcomes [7, 34]. Efsen et al. reported the upregulation of CX3CL1 and CX3CR1 in hepatocytes and human HCC cell lines during liver injury [35]. Matsubara et al. reported a positive correlation between the expression of CX3CL1 and CX3CR1 and both disease-free survival (DFS) and OS [7]. Their study also revealed that activation of the fractalkine-CX3CR1 axis inversely correlates with increased cell proliferation and tumor differentiation in HCC, potentially reducing survival rates. Sun et al. demonstrated that CX3CL1 can promote the invasive and migratory capabilities of HCC cells through the Src/PTK2 signaling pathway and indicated that the overexpression of CX3CL1 is directly associated with the spinal metastasis of HCC in mouse models [36]. These variations may be explained by differences in tumor biology, gene expression profiles, and inflammatory mediators present in different cancer types.

To further explore the mechanisms by which CX3CL1 influences the tumor microenvironment, we conducted qPCR and ELISAs. Our qPCR analysis revealed that CX3CL1 overexpression significantly upregulated the expression of MDSC-recruiting factors, including CXCL13, CXCL2, and IFN-α. Furthermore, the ELISA results corroborated these findings by demonstrating a marked increase in the secretion levels of IFN-α and CXCL2 in the cell supernatants. These results suggest that CX3CL1 may facilitate the accumulation of MDSCs within the HCC microenvironment through these factors, thereby promoting immunosuppressive mechanisms. This observation aligns with the literature, identifying CXCL2 upregulation as a critical pathway for MDSC recruitment in various tumor types. Notably, we observed a significant increase in IFN-α expression, specifically in HepG2 cells, while CXCL2 was consistently upregulated across all the examined cell lines. Such variations may reflect the distinct regulatory properties of CX3CL1 in different cellular contexts, underscoring its complex and multifaceted roles.

This evidence identifies CX3CL1 as a potential therapeutic target. Inhibition of CX3CL1 or its downstream signaling could reduce MDSC-mediated immunosuppression and increase the efficacy of immunotherapies. For example, recent studies have shown that inhibiting the CX3CL1‒CX3CR1 axis, particularly with drugs such as the Src inhibitor saracatinib, can block the chemotactic effect of CX3CL1 and reduce tumor metastasis in other cancers [37, 38]. Given that HIF-1α regulates the recruitment of immune cells through the CX3CL1-CX3CR1 axis [9], blocking the HIF pathway, which regulates immune cell recruitment through CX3CL1, may promote the antitumor immune response by decreasing MDSC and TAM recruitment while increasing the number of CD8 + T cells and NK cells [39].

This study has several limitations, including a small sample size, the constraints of a retrospective design, and potential biases in sample selection. While we observed a significant correlation between elevated CX3CL1 expression in HCC and increased MDSC infiltration, the precise mechanisms underlying CX3CL1-mediated MDSC recruitment remain to be fully elucidated. Nonetheless, the clinical relevance of this phenotype underscores the importance of our findings. Future studies focused on the CX3CL1‒CX3CR1 axis in the tumor microenvironment, including blockade experiments, are needed to validate these mechanisms.

In summary, our study highlights the potential role of CX3CL1 in shaping the tumor microenvironment by recruiting MDSCs and possibly contributing to immune evasion in HCC. Targeting this axis may provide novel therapeutic strategies to promote antitumor immunity and improve clinical outcomes for patients with HCC.

## Supplementary Information

Additional file 1.

## Data Availability

The original contributions presented in the study are included in the article/supplementary material. Further inquiries can be directed to the corresponding authors.
