# Supplementary material for: HCC-derived CX3CL1 affects hepatocellular carcinoma prognosis and CX3CR1 + MDSC infiltration
Source: Eur J Med Res. 2025 Mar 6;30:153. doi: 10.1186/s40001-025-02410-z (PMC11884201; doi:10.1186/s40001-025-02410-z)
Supplement: Supplementary file 1 — Additional file 1. [file 40001_2025_2410_MOESM1_ESM.docx]

| 1. primer sequences |  |
| --- | --- |
| 1. GAPDH-f | 1. GAAGGTGAAGGTCGGAGTCA |
| 1. GAPDH-r | 1. GAAGATGGTGATGGGATTTC |
| CX3CL1-f | 1. ACCACGGTGTGACGAAATG |
| 1. CX3CL1-r | TGTTGATAGTGGATGAGCAAAGC |
| 1. CXCL5-f | AGCTGCGTTGCGTTTGTTTAC |
| 1. CXCL5-r | TGGCGAACACTTGCAGATTAC |
| CXCL13-f | GCTTGAGGTGTAGATGTGTCC |
| 1. CXCL13-r | CCCACGGGGCAAGATTTGAA |
| 1. DDR2-f | GCTATATGCCGCTATCCTCTGG |
| 1. DDR2-r | ACTCTGACCACTGACTGGAAG |
| 1. STAT3-f | CAGCAGCTTGACACACGGTA |
| 1. STAT3-r | AAACACCAAAGTGGCATGTGA |
| 1. IFN-α-f | AATACAGCCCTTGTGCCTGG |
| 1. IFN-α-r | AGCAGGGGTGAGAGTCTTTG |
| 1. CXCL5-f | AGCTGCGTTGCGTTTGTTTAC |
| 1. CXCL5-r | TGGCGAACACTTGCAGATTAC |
| 1. CXCL2-f | TTCACAGTGTGTGGTCAACAT |
| 1. CXCL2-r | TCTCTGCTCTAACACAGAGGGA |
